# Both Physical and Virtual: On Immediacy in Esports

**DOI:** 10.3389/fspor.2022.883765

**Published:** 2022-05-09

**Authors:** David Ekdahl

**Affiliations:** ^1^Department of Sports Science and Clinical Biomechanics, University of Southern Denmark, Odense, Denmark; ^2^Department for the Study of Culture, University of Southern Denmark, Odense, Denmark

**Keywords:** esports, immediacy, physicality, virtuality, embodiment, video games, sports

## Abstract

This article strives to make novel headway in the debate concerning esports' relationship to sports by focusing on the relationship between esports and physicality. More precisely, the aim of this article is to critically assess the claim that esports fails to be sports because it is never properly “direct” or “immediate” compared to physical sports. To do so, I focus on the account of physicality presented by Jason Holt, who provides a theoretical framework meant to justify the claim that esports is never properly immediate and therefore never sports. I begin by motivating Holt's account of physicality by contrasting it with a more classical way of discussing physicality and sports, namely in terms of physical motor skills. Afterwards, I introduce Holt's account of physicality as immediacy and engage with its assumptions more thoroughly to problematize the claim that esports is fundamentally indirect. Lastly, I argue that the assumption that esports necessarily lacks immediacy is based on a narrow understanding of body and, consequently, of space. In response, I offer a different way of thinking about body and space, focusing on the subjective, bodily engagement of the esports practitioners with their practice, whereby physical space and virtual space can be appreciated as immediately interconnected during performance in a hybrid manner. In providing such an account, the article contributes directly to the broader, growing discussion on the relationship between physicality and virtuality in an increasingly digital world.

## Introduction

In 2020, esports became a billion-dollar industry. With emerging markets increasingly embracing this trend, and with the mounting dominance of digital culture more broadly, its growth shows no sign of stopping any time soon. Combined with the name itself, e-sports, the phenomenon has sparked an enduring discussion about its relationship to sports. To claim that at least some form of esports can qualify as sports is what Jason (Holt, 2016, p. 6) has labeled the “cybersport thesis” – a label I adopt in this paper.^1^ The cybersport thesis has in various forms provoked responses from commentators (Burns, 2014; Johan Cruyff Institute, 2017), journalists (Young, 2016), professional athletes (Kim and Lee, 1960) and even late-night comedians (Jimmy Kimmel Live, 2015). Within the gaming milieu itself, there has also since the early days of modern esports existed an on-going discussion about the cybersport thesis, including whether all forms of esports practice merit a sports status evenly, as well as whether a sports status is at all relevant to esports (Taylor, 2012, p. 51).

Philosophically informed explorations of what constitutes sports, and whether esports can fit into such definitions, have also found their way into the discussion (Jenny et al., 2017; Rosell Llorens, 2017; Hallmann and Giel, 2018; Parry, 2019; Naraine, 2021). These contributions have^1^ raised important points about the nature of esports, especially where and how it succeeds or fails to live up to varying concepts of sports. Of course, one methodological limitation of holding up esports to some stipulated definition of sports in this way is that many different conceptions of sport exist (Jeu, 1972; Suits, 1988, 2007; Guttmann, 2004; Barbu et al., 2020; Sanz de la Garza and Adami, 2020; Borge, 2021). Even when esports is held up to internationally recognized standards of sports, such as the Olympics (Parry, 2019), conflicting philosophical definitions of such sports also exist, some of which are perfectly capable of allowing esports a sports status (Møller, 2009).

While the discussion surrounding the cybersport thesis provides a framework for this article, my goal here will not be to resolve it. I am, in this context, less focused on what makes anything a sport, than I am with our understanding of esports. After all, even if a proper or sufficient concept of sports existed and could be found, this alone does not suffice to engage with the cybersport thesis; one must also have a proper concept of esports. This article seeks to make novel headway in the discussion surrounding the cybersport thesis by focusing specifically on a dimension of sports that is broadly assumed to be essential to the concept itself, and which has become a central theme of the discussion surrounding esports: physicality. “Physicality” can, like sports, of course mean different things, e.g., physical exertion, physical risk, or physical health, all of which have been explored by other researchers within the context of esports (Bayrakdar, 2020; Emara et al., 2020; Kocadaǧ, 2020; Haupt et al., 2021; Ketelhut et al., 2021; Giakoni-Ramírez et al., 2022; Wall Tweedie, 2022). Instead, I will focus specifically on the claim that there exists a gap between the physicality and virtuality of esports performance; that “where” an esports practitioner performs is only indirectly related to “where” the result of her performance occurs. That is to say, the aim of this article is to critically assess the claim that esports performance is fundamentally “indirect”. To critically assess this claim, my focus will be on the work of Holt (2016), who, in defining physicality in the sporting sense of the word as immediacy, develops a theoretical framework meant to show why no performing esports practitioner is ever directly or immediately related to their virtual playing field.

To critically assess this, I will begin by providing a background to the discussion of physicality in esports, which will help motivate the significance and novelty of Holt's account. I do this by discussing how the relevance of physicality to sports has often been presented, namely in terms of motor skills. I will here argue that, even when granting the necessity of physical motor skills for something to be sports, this nevertheless fails to convincingly dismiss the cybersport thesis. Second, I introduce Holt's account of physicality in sports, and show how he deploys this in a much more encompassing attack on the cybersport thesis in what I label an “argument from immediacy”. Third, I critically assess three ways of justifying Holt's argument from immediacy – all of which with varying degrees of focus are noted by Holt himself. I conclude that none succeeds in decisively justifying the argument from immediacy without ending in metaphysical dogmatism about virtuality. Fourth, I examine the roots of Holt's account, specifically the narrow understanding of body and space that it seems to depend on. In contrast, I introduce the concept of the “body subject” and show how this concept allows us to appreciate the immediacy that can characterize esports performance. Finally, the limitations, as well as the theoretical and practical implications of this study are considered, including the direction for future research on the relationship between physicality and virtuality in esports.

## Physicality as Motor Skills

To appreciate the novelty and impact of Holt's account of physicality, it is relevant to begin by considering how physicality has often been discussed within the context of the cybersport thesis, namely in terms of motor skills (Hilvoorde and van Pot, 2016; Jenny et al., 2017; Rosell Llorens, 2017; Parry, 2019). To begin with, motor skills have been described as falling on a spectrum between fine and gross, a distinction spelled out by Haibach-Beach et al. (2011) with reference to the nature of the physical movements and the width of muscle groups deployed. Fine motor skills can be understood as bodily movements that primarily involve control and accuracy, with a narrow range of muscle groups deployed, such as we might find in dart games. Gross motor skills, in comparison, are physical movements that involve larger muscle groups or whole-body activity, such as we find in running or swimming. Of course, no exact delineation between the two forms of motor skills can be drawn and often sports will involve both. Nevertheless, this does not bar us from distinguishing between them pragmatically (Parry, 2019, p. 13), including when assessing the kind of motor skills that do or do not characterize esports performance.

### Fine Motor Skills

Fine motor skills are arguably the least controversial, physical aspect of much esports. In many of the most popular and enduring forms of esports, the practitioners must move their arms and fingers rapidly. In the real-time strategy (RTS) game StarCraft II, professional player Park Sung-joon famously reaches over 800 APMs (actions per minute), or more than 13 actions per second, during competitive performance. Such speed alone is impressive, yet an overly narrow focus on speed alone (cf. Parry, 2019, p. 12) neglects the degree of precision and fine motor control involved. Stressing exactly these elements, Lowood (2007), within the context of RTS games like StarCraft II, notes

[t]he criticisms leveled at RTS games of reducing strategy play to mindless mouse-clicking misunderstands the denigrated “clickfest” or “button-mashing” by missing the connections between mastery of syntax and strategy, both invisible on the screen (Lowood, 2007, p. 94).

The fine motor movements needed in many esports games are goal-oriented and precise, requiring a significant amount of control and focus, and with a single miss-click potentially having disastrous effects in-game (as well as, possibly, for the practitioner's career). As Taylor points out:

You may know what you should do, but your reaction times and your ability to carry out in concrete ways the strategic decisions you make are key. Language like “flailing” or “button mashing” – where the link between what you see and want to act on runs up against the actual ability to act – highlights how central control of one's body is for computer gaming (Taylor, 2012, p. 53).

Notably, because of the extreme degrees of speed and precision involved, esports practitioners rarely perform professionally for very long, often retiring by their mid-twenties (Suncho, 2022). However, while the intricacy of the fine motor skills pertinent to esports are important to emphasize when considering the physicality of the phenomenon, they are typically not the primary category of motor skills that critics of the cybersport thesis stress when arguing that esports is not physical.

### Gross Motor Skills

When assessing the kind of physicality necessary for something to be sports, gross motor skills are often emphasized (Loy, 1968; Hemphill, 2005). Jenny et al. thus argue that

eSports only necessitate fine motor movements as a player manipulates a handheld controller. Many games, including those such as Jenga and eSports, only involve fine motor skills for successful completion, which does not meet the condition of physicality within common definitions of sport (Jenny et al., 2017, p. 10).

The picture of esports practitioners is at times that of sedentary performers using their gaming equipment with some localized speed and accuracy – “[f]rom my $399 gaming throne, I operate my console so as to achieve digital effects on a screen”, as Parry (2019, p. 12) remarks. There are at least two issues with such a view of esports performance when trying to dismiss the cybersport thesis. First, even relatively sedentary, forms of pro gaming where practitioners are seated and use conventional PC-setups have, especially at top levels of esports performance, been noted by researchers to correlate with control and engagement of the broader physical body in more nuanced ways than one might first assume. From her work with actual esports practitioners, Taylor notes:

We can also see the broader ways players' bodies are enlisted. Tense shoulders, focused visual attention, “on point” posture, complex cognitive engagement, and stillness in the body except for the key interface points (eyes, hands, and even feet) all speak to the ways the body is not only always present in computer game play, but indeed the ways the mastery of the body is crucial in pro gaming, much like in traditional sports (Taylor, 2012, p. 53).

Moreover, Ekdahl (2021) has shown that a continuous sensitivity to and control of the physical body during performance can be central to high-ranking esports practitioners, just as Witkowski (2012) has emphasized the importance of breath-control. It might here be objected that elements like posture, bodily sensitivity, and control, including breath control, are too far removed from the kind of gross-motor activity required in sport. Regardless of whether one views shooting as a sport, consider in this context Parry's argument for the central relevance of whole-body control to Olympic shooting:

[…] in regard to shooting, it is false that the required movement involves merely squeezing a trigger. This fails to take into account the total-body control required of a shooter, including balance, stance, rifle hold, controlled breathing, etc., all of which contribute directly to the outcome (Parry, 2019, p. 9).

Barring whether whole-body control is an especially good way of delineating Olympic sports, as it risks leaving many types of paralympic sports outside the realm of sports, it is unclear why the above attention to detail in terms of bodily engagement cannot also be extended to how we view esports performance. As Naraine (2021, pp. 39–40) points out, “Just as shooting is more than squeezing a trigger, so, too, is esports”.^2^ This is especially relevant given that researchers working with actual esports practitioners have repeatedly stressed similar dimensions as contributing directly to the outcome of the performance. Second, assuming nonetheless that accounts of whole-body engagement such as these do not show that some forms of traditional, more sedentary esports practice are sufficiently “motor-gross”, one could point to the growing amount of esports practices that have begun relying on more whole-body systems, such as various forms of VR^3^ and AR^4^ esports, as well as so-called “physical esports”, e.g., virtual bicycle racing. Similarly, an example of more whole-body oriented gaming, as noted by Jenny et al. (2017), are MBVGs – motion-based video games, such as those relying on systems like the Xbox 360 Kinect or the PlayStation move (Kinect for Xbox 360, 2022; PlayStation, 2022) – which exactly “track gross motor physical body movements” as an essential part of the performance^5^ If all that is missing for esports to be properly physical are gross motor skills, at least some esports practices seem to have made it to the promised land.

Either way, neither the requirement of physicality as fine nor gross motor skills decisively refute the cybersport thesis. The scene is thus set for introducing another much more fundamental way of drawing the distinction between esports and sports in terms of physicality – namely with reference to the immediacy between practitioner and playing field.

## Physicality as Immediacy

In his contribution to the debate surrounding the cybersport thesis, Holt (2016), develops a theoretical framework intended to show that there is a fundamental gap between physicality and virtuality in esports.^6^ To show why esports or “cybersports” can never be properly physical, Holt introduces a framework based round two concepts: “domain of execution” and “domain of application” (Holt, 2016). These are defined by Holt in the following manner:

The domain of execution is subject-specific, a matter of where the execution occurs; by contrast, the domain of application is object-specific, where the action's outcome is meant to obtain (Holt, 2016, pp. 8-9).

I will refer to this framework of domain of execution and domain of application as *Holt's theoretical framework*. In further clarifying it, Holt (2016, p. 8) contrasts the case of a face-to-face conversation with that of a telephone conversation, noting that in the case of a face-to-face conversation, there is a general overlap between domain of execution and domain of application, whereas a telephone conversation involves, Holt argues, a significant spatial difference between these domains. An overlap in the domains of execution and application is then presented by Holt (2016, p. 9) as an essential feature of sports: Where the subject is acting must also be where the results of their activity occur. For Holt, this is where esports can be seen to fall outside the world of sports. According to Holt, there is something qualitatively different about the way esports practitioners engage with their playing field, over and above the motor skills required, compared to how purely physical athletes engage with theirs. In the context of Holt's theoretical framework, there is something qualitatively different about the relationship between domain of execution and domain of application in esports. For Holt, this difference concerns a lack directness or overlap in the relationship between subject and playing field in esports: An esports practitioner simply cannot engage directly with their domain of application because the domain of application as a space is removed from the subject's space of execution. That is to say, according to Holt, esports necessarily involves separate domains of execution and application:

Cybersport exhibits, as it must, a technologically realized separation between domains of execution and application that are in principle disjoint. In ordinary sport, the domains coincide on the actual playing field: where I take the shot is also, unremarkably, the same realm in which I aim to score a goal. But this cannot be the case for cybersport, which by definition requires that skills be executed in one domain, which is actual, and then transposed into a virtual one (Holt, 2016, p. 9).

I will refer to this argument as the *argument from immediacy*. Holt's argument from immediacy echoes a point also emphasized by Hutchins (2008) who, in a similar vein, has noted that the media aspect of esports, the fact that it is digital or virtual, can be leveled as a point against the cybersport thesis. That is to say, the mere presence of virtuality as part of the performance can be seen as a disqualifying element in and of itself for something to be a sport. Holt's description of the essential gap between the domains of execution and application in esports does allude to something seemingly true about esports performance: The esports practitioner must perform from one domain of execution – for instance, manipulating a set of gaming peripherals in front of a monitor, whereas the results of these activities occur “virtually” in a separate domain of application. In this way, Holt's argument from immediacy is especially significant and novel because, in delineating what counts as sport, Holt's focus is uniquely on the kind of space that a competitive practice takes place in, including the performing subject's relationship to this space. Because of this, Holt's approach to the cybersport thesis is at the same time a way of bringing to the fore the issue of virtuality in sports in an increasingly digital age.

Notably, Holt's critical account of the spaces of esports as indirect spaces can be seen as one of several, similar contributions that jointly make up a broader, multidisciplinary criticism of virtual spaces. As an example, from the field of psychiatry, Fuchs (2014) has argued that virtual spaces at best allow only for an indirect or quasi-real engagement which consequently prevents immediate, empathetic interaction with others. Similarly, sociologist Hartmut Rosa (2019, p. 92) has described virtual spaces facilitated by computer screens as something that convey significance only indirectly or “symbolically”. And, most famously perhaps, philosopher Hubert Dreyfus, raised serious skepticism about the potential of virtual spaces, often emphasizing exactly their “indirectness” (Dreyfus, 2008, p. 113). Holt's account is, however, the first and most accessible attempt at integrating the supposed indirectness of virtual spaces into the discussion surrounding the cybersport thesis. For this reason, it is Holt and especially his argument from immediacy that are here focused on – an argument that will undoubtedly resonate with many; seemingly managing to capture something that essentially distinguishes physical sports from esports. Indeed, Holt even describes his argument from immediacy as

[giving] a theoretical basis for the intuition that it is the virtuality of the video game environment, in contrast to the physicality of the traditional sport environment, that prevents inclusion of videogames in the sport category (Holt, 2016, p. 9).

As Parry concurs:

[Holt's account] is one way of clarifying, specifying, exhibiting the lack of direct physicality in computer games, that argues against its status as sport (Parry, 2019, p. 12).

The argument from immediacy is, critically, not based on the degree of complexity or skillfulness of the esports practitioners' physical performance, but on a supposed gap between the esports practitioners' physical performance and their virtual domain of application. In this way, even if a physical sport and a digital equivalent had a complete motor skill overlap and required symmetrically similar forms of motor engagement, the digital equivalent would never in the sporting sense of the word be physical on Holt's account, because the domain of execution and the domain of application would not overlap. Holt openly commits to this:

Even a cybersport identical to an actual sport in every detail of skill execution – as with, for instance, the holodeck in Star Trek's fictional universe – will fail to be sport for this reason (Holt, 2016, p. 9).

Holt concludes his analysis of esports and physicality by noting that

[c]ybersport fails to be sport only if […] the difference in their domains of application is a significant and principled one […] (Holt, 2016, p. 12).

A consequence of the definition of physicality as immediacy, compared to the definition of, for instance, physicality as motor skills, is therefore that the stakes are now much higher in terms of the cybersport thesis. Whereas an argument from gross motor skills, as an example, might be seen as leaving some (but not necessarily all) forms of esports out of the fold of sports, the argument from immediacy, if successful, leaves all esports practices qua virtuality out of the fold of sports. Conversely, if the gap in immediacy between domain of execution and domain of application in esports is unfounded or hollow, this would seem to leave the door ajar for esports to be, in Holt's sense of the word, physical – and, possibly, sport. In this context, I contend that there are at least two fundamental issues with Holt's argument from immediacy, which will structure the remainder of this article. First, no sufficient justification for the gap between domain of execution and domain of application in esports is provided by Holt outside of metaphysical dogmatism about virtuality. Second, Holt's theoretical framework itself, which grounds the argument from immediacy, has at its roots a particularly narrow understanding of body and, consequently, of space. Let us begin with the first issue.

## Justifying the Argument From Immediacy

Before beginning, it is important to de-couple two related but distinct elements of Holt's argument from immediacy. The first is that esports involve both physicality and virtuality. This is entirely uncontroversial, and on its own adds nothing to the discussion around the cybersport thesis. However, the second element is that, because esports involve both physicality and virtuality, there cannot be an immediate overlap between domain of execution and domain of application as they are essentially separate in esports. It is the second element that I will challenge.

A problem in this context is that, while Holt alludes to different distinctions between esports and sports, no one clear argument is provided for the essential separation or distinction in domains between physicality and virtuality in esports. At times, the essential distinction is even presented by Holt as if real prima facie, and even as self-evidently “metaphysical” (Holt, 2016, p. 9). But the question remains what we mean when we say that physicality and virtuality are metaphysically distinct. Was Descartes (2013) limited by his place in world history when he only identified matter and mind^7^ as distinct substances? Should we also include virtuality as a separate substance? Yet even Descartes allowed for direct interaction between his disparate substances. And, indeed, things can be metaphysically distinct without necessarily being indirectly related. A baseball bat is metaphysically distinct from the ball it strikes, yet these are arguably not indirectly related. The core question is then: In what way are we justified in insisting on a metaphysical gap between physical and virtual that prevents overlaps in domains of execution and application, and thus prevents immediacy. Here, it will not do to simply reiterate the intuition that sports is direct because it is through-and-through physical compared to esports, as this again just begs the question: Why are only physical domains of execution and application able to stand in a direct relation? Consider again Descartes who ascribed essential attributes to his metaphysically distinct substances to distinguish between them. If the argument from immediacy is right, what then are the “attributes” or features of virtuality at stake in esports that might justify this metaphysical gap in immediacy?

Before considering three ways of justifying the gap between domains of execution and application in relation to esports that, with various degrees of priority, are mentioned by Holt, I want to make two preliminary notes. First, to critically assess the argument from immediacy, I will from the outset have to bracket the assumption that physicality and virtuality are metaphysically distinct in the way argued by Holt. I do this to avoid the justification becoming tautologic, i.e.: “Virtual and physical domains in esports are metaphysically distinct in a way that allows no immediacy because one is virtual, and one is physical”. If the argument is right, it must be possible to justify this without the justification becoming circular. Second, as I discuss more thoroughly the understanding of body and space that is implied by Holt's theoretical framework later on, I will, for now, leave these concepts out of my assessment of Holt's argument from immediacy.

### Esports' Domains of Application Are Too Technological

One way to differentiate the relationship between domain of execution and domain of application in sports contra esports is with reference to the technology esports depends upon. In this context, a general skepticism toward the adverse relationship between too much technology and the authenticity of sports is not uncommon – an obvious example is the discussion surrounding the place and role of Video Assistant Referee systems or “VAR” systems in football (Olkhov, 2021; Tamir and Bar-eli, 2021).

In the context of the cybersport thesis, Parry (2019, p. 8) draws a parallel between why esports and motor-boating both fail to qualify as (Olympic) sports, arguing that that the reason we can accept, e.g., sailing but not motorboating as an Olympic (read: real) sport is because motorboating is too much of an engineering feat and thus too far removed from the immediate, human element. Intended as an analogy to esports, this argument can be read in one of two ways. Either the argument is that when engineering or technological feats impose an overly effective advantage of one performing athlete over another, e.g., when one motorboat functions better than the others, then the practice is insufficiently human. This analogy, however, does not apply very neatly to most esports practices, where non-peripheral hardware (e.g., the CPU, GPU, RAM, hard-drive, monitor, etc.) is unlike the engines and engineering feats of motorboating. This has to do with *relationality*: Because a particularly powerful computer might grant an advantage to an individual esports practitioner (e.g., faster loading, less lag, higher frame rate and resolutions, etc.), the hardware of a particular esports practice must typically be identical across the field of performers during competitions.

It is true that the players of certain platforms, are allowed to perform on their own gaming peripherals (usually mouse, keyboard, headset, and cords), but these peripherals are much more accurately aligned with the sort of personal equipment found in traditional sports competitions than the engines of motorboating. The players that are allowed to use their own peripherals do so because the peripherals are not seen as playing the game for them or giving them an unfair advantage, but rather as tools facilitating a complex and very personal skillset that involves bodily sensitivity and preference when it comes to equipment (Taylor, 2012; Ekdahl, 2021).

If, alternatively, the analogy to motorboating is meant to show that technology, in some indeterminate quantity and/or quality, bars something from being sufficiently human (and thus a real sport) altogether, this brings the argument back to the discussion of domains of execution and application, and comes closer to a sentiment found in Holt's claim that

[i]t is technology that often facilitates a useful separation between these domains, and we might even define technology in terms of such separation (Holt, 2016, p. 9).

Yet, sports has always uncontroversially revolved around technology and can often even be appreciated as physical mastery of various forms of technology. The claim must then be that some forms of technologies by themselves invoke a gap between domains of execution and application, whereas others do not. What is it especially, then, about esports that technologically separates these domains?

One obvious argument here is that the technologies of esports are unlike the technologies of physical sports because they are virtual. That is to say, the pertinent claim would not be, contrary to Holt's definition above, that there must be an adverse relationship between technology and immediacy. Instead, the claim would be that it is the virtual technologies of, e.g., esports that has an adverse effect on immediacy. This is formulated by Holt as a question of technological transpositionality, with esports being seen as involving a transposition of input in one domain to an output in separate domain (Holt, 2016; see also Parry, 2019, p. 12). However, the fact that esports involves technological transpositionality alone cannot carry the argument that esports' virtual domain of application are separate from the practitioners' physical performance. Technological transpositionality is uncontroversially an integral part of numerous forms of sport already. A cyclist's activity while biking is plainly transposed through a particular technology (a bike) to produce a unique output (the bike moving forward) – an output which cannot be reproduced in the same manner without the bike being present. Thus, it is the virtuality of esports technology that must carry the argument that the transpositionality from domain of execution to domain of application is indirect. But, as already noted, it will not suffice to simply point to the virtual dimension alone as prima facie metaphysically distinct from the physical domain when arguing for the indirectness of esports – as this already presupposes the gap it purports to show. We are still owed an explanation of what it is about the virtual domains of esports that make them fundamentally indirect.

### Esports' Domains of Application Are Representational

One categorical difference one might emphasize, including in the context of virtual transpositionality, is that the esports practitioners' physical performance are transposed onto domains that are only indirect representations as they are only images (Holt, 2016, p. 11). As Parry remarks:

Computer gamers are not, coddled in their special armchairs, direct competitors. They are distanced, image-manipulating remote-controllers (Parry, 2019, p. 12).

On such accounts, the domains of application in esports, unlike the domains of application in conventional sports, are fundamentally representational – they are mere phantasms that the practitioners' physical body can consequently only lean back in their chairs and manipulate indirectly. We find a similar sentiment in Hartmut Rosa's brief discussion of screens more generally, which he describes as something that

[…] provide access to the world only as mediated via symbols and through a digitalized filter of hard, rigid always identical surfaces (Rosa, 2019, p. 92).

There are several objections one might raise to such sentiments. To begin with, in the context the screens of esports specifically, it is worth noting preliminarily that the universal need for screens in esports will likely eventually become a thing of the past. As virtual technology continues to develop, so, too, the virtual technologies deployed in esports will – moving further and further away from a need for screens altogether (Chalmers, 2022, pp. xii, 208).

Bracketing such futuristic speculation, it is perfectly possible to be critical of the notion itself that images in any performance in and of themselves prevent overlap between domains of execution and application. Consider a professional archer that develops an eye disease like optic atrophy, but regains normal, functional vision by using a form of so-called “smart glasses” (Home, 2022), which deploy a high-speed camera that captures what she is looking at, and projects it as a live video feed in front of her eyes. Provided the camera in this hypothetical bestows no performative advantage, and that, to the archer, there ends up being no relevant difference between having her normal sense of sight and using smart glasses, do the images themselves bar an immediate relationship between domain of execution and application? One might interject here by noting that, in this case, both domain of execution and domain of application stand in direct relation because they are both physical, regardless of the presence of digital glasses. Barring that this already again assumes only physical domains can overlap, once we admit to this, we must first acknowledge that the digital images do not by themselves invoke a chasm between domain of execution and application.^8^ Conversely, one might bite the bullet and agree that there is no longer an immediacy between domain of execution and application for the archer as her performance now involves images and is thus indirect.

We should in this context be critical of the idea that images can only be indirectly engaged with simply because they are images – an issue that has likewise been explored by other scholars (Ihde, 1990; Introna and Ilharco, 2006; Osler, 2021; Chalmers, 2022). It is true that, in the context of esports, “mini-maps” and similar indirect kinds of representations that provide more abstract information about the digital playing field can be an integral part of performance. However, just as we can unproblematically differentiate between indirect representations and direct presentations in physical domains (e.g., a map over New York City compared to the physical city itself), it is not clear why we cannot do so in the context of virtual domains, too.

Barring phenomena like mini-maps, esports practitioners' immediate point of the view as they engage their virtual domain, whether it be first-person or not, is not self-evidently a question of seeing a series of indirect images or symbols on a screen to be manipulated. One way of appreciating this is to understand that, to a capable esports practitioner, perceiving and interacting with the virtual images of esports just means perceiving and interacting with the virtual playing field itself (Ekdahl and Ravn, 2019): There is no disparity between the images on the screen and the perceived virtual domain. “When I see Pac-Man on the screen, I'm really seeing Pac-Man. The screen enables me to see him”, as Chalmers (2022, p. 208) notes. Even if facilitated by digital images on a screen, “[t]he eSports practitioner must lose some indeterminate part of his or her sense of being in front of a monitor, peering into a digital world […]”, as Ekdahl and Ravn emphasize (Ekdahl and Ravn, 2019, p. 6). Moreover, because of the degree of interactivity found in many of the virtual domains of esports, Klevjer (2012, p. 28) has highlighted that mastering virtual gaming often involves a new “perceptual ecology” for the practitioner. This is to say that it involves learning to navigate the competitive, virtual domain exactly not as a series of indirect images, but as something with immediate, perceptual and practical significance for the practitioner.^9^ In short, the fact that esports practices (for now) revolve around screens and images fails to persuasively show that domain of execution and application cannot overlap in said practices.

### Esports' Domains of Application Are Not Real

Before returning more closely to the meaning of body and, consequently, of space, integral to Holt's theoretical framework, I will consider one final, encompassing way of justifying the argument from immediacy.

It is significant that Holt, in his criticism of the cybersport thesis, deploys the example of the holodeck from Star Trek (see Section Physicality as Immediacy) to drive home his point that virtuality is metaphysically distinct from physicality in a way that allows no immediate overlap in domains. This is so because the holodeck is a room capable of virtually replicating physical reality. If the crewmembers aboard the U.S.S. Enterprise, where the holodeck can be found, engaged with any conventional sport like football inside it, it would not, on Holt's account, properly be sport because the domain of application is still at its core virtual. This is independent of whether the holodeck allows for the same detail in skill execution as a physical domain, including, notably, if it replicates things like tactility, solidity and even mass with perfect realism. Yet, if a digital system like the holodeck could in theory replicate a physical sport down to the point where the sport is physically indistinguishable from a non-simulated sport to the performer, why is one but not the other direct? If the holodeck could digitally replicate the entire physical domain of a runner's track, why are practitioners running inside the holodeck not immediately related to their domain of application?

Given that neither technological transpositionality nor the supposed representationality of virtual domains can carry the argument from immediacy, and given that, even if virtuality could perfectly mirror physicality, this would still not be something on Holt's account that a practitioner can really engage with directly, this seems to leave us with only one way of differentiating a physical domain from a virtual domain: reality. Of the three ways of justifying the argument from immediacy, Holt most clearly emphasizes the absence of this dimension of virtuality:

[…] we are dealing with virtual domains in that where play eventuates is non-actual, depicted on a television or computer monitor; it is perhaps a digital representation of, and might possibly be mistaken for, the real world, but is not actually itself the real world (Holt, 2016, p. 6).

The conclusion seems to be that the virtual domains of esport no more than the virtual domains of the holodeck allow for immediacy because they are fundamentally not real. The obvious interjection here is: Why not? Toward the end of his treatment of the cybersport thesis, Holt writes:

All along I have assumed that there is a significant and principled distinction between the actual world and various virtual worlds, an assumption whose roots are philosophically very deep […] (Holt, 2016, p. 12).

In this way, it is a particular metaphysical assumption about the non-reality of virtuality that, more than anything, drives the argument from immediacy: The virtual domains of esports are not real, and thus there can be no overlap in domains of execution and application. Holt's argument from immediacy therefore depends fundamentally on a refusal of what we might here call “virtual realism”: that virtual phenomena can be real even if they are not physical; that physicality and reality are not one and the same thing.

Despite Holt's insistence on the philosophical roots for his principled distinction between physical and virtual, it is worth noting that equating reality with physicality is not itself unproblematic, as reality has over the course of millennia been defined by philosophers in many ways – often prior to the advent of virtuality. Following Chalmers' (2022, pp. 105–116) recent analysis of the relationship between reality and virtuality, we can surely acknowledge that the virtual domains of esports are both casual and mind-independent – themselves sufficient criteria for much of what we uncontroversially consider real. In this way, without any further support, basing the argument from immediacy on an assumption that virtuality is not real only really amounts to metaphysical dogmatism. In sum, outside of metaphysical dogmatism about virtuality, we have so far failed to justify the metaphysical gap between domain of execution and domain of application in esports.

## Bodily Immediacy in Esports

Fortunately, metaphysical dogmatism about virtuality does not have to be the end of the story about immediacy in esports. As I will argue, Holt's theoretical framework, which grounds his argument from immediacy, depends not just on a metaphysical assumption about the non-status of virtuality, but also on a narrow understanding of body and, consequently, of space.

### The Body Subject

To appreciate more clearly Holt's understanding of the body, consider again the meaning of “domain of execution”. As noted, Holt defines this domain as “subject-specific, a matter of where the execution occurs” (Holt, 2016, p. 9). Two elements stand out in this definition: the subject, and its activity (the “execution”). The idea seems to be that “the subject is where the subject does”: A football player kicks a ball past the opposing goalie and scores a goal; where the subject is, is where the subject is kicking said football – which, in this case, is the space of the football field. On Holt's account, there is therefore here overlap between domain of execution and domain of application. So far, so good. Let us now consider the case of a performing esports practitioner.

An esports practitioner is seated in front of a screen, manipulating her physical hardware with speed and precision, her in-game avatar nimbly dodging out of the way of enemy attacks in close correlation with the practitioner's physical performance – the audience at the edge of their seats. For Holt's argument from immediacy to work, the domain of execution of the esports practitioner can only be where she is manipulating keyboard and mouse, and not also where she is manipulating her avatar. But why should “where the esports practitioner is acting” not also uncontroversially include her virtual performance? The reason this is not an available option on Holt's account is inevitably connected to the account's implicit understanding of “where the subject is”, specifically as “where the subject's physical body is”. In this way, Holt's argument from immediacy can be appreciated as an argument from *bodily immediacy*: The reason the activity of the esports practitioner (‘where she is”) cannot include the virtual activity of her avatar, is that only the activity of the physical body is properly the activity of the subject. Barring how well this argument works in the context of VR and AR esports, the point is that the activity of the esports practitioner is never “where” the avatar is, as the body (a physically extended object) ends before the virtual space begins.

This is, however, a narrow understanding of body. In fields ranging from philosophy (Merleau-Ponty, 1983, 2013; Leder, 1990; Gallagher, 2005; Zahavi, 2010; Taipale, 2014; Gallagher and Zahavi, 2020), psychology (James, 1948; Gibson, 1983, 2014; Kyselo, 2014; Körner et al., 2015; Zatti and Zarbo, 2015), biology (Varela et al., 2017), to sports science (Breivik, 2008; Allen-Collinson, 2009; Allen-Collinson and Hockey, 2009; Legrand and Ravn, 2009; Ravn and Christensen, 2014; Aggerholm and Højbjerre Larsen, 2017; Ravn and Høffding, 2017; Heath and Larsen, 2022; Mudyahoto et al., 2022), there has over the past century been a growing insistence that we fail to fully capture the meaning of the body when we treat it as a physical object alone. We not only have a physically extended body that might undergo physical relocation as it moves through space, but we are also at the same time inevitably experiencing and interacting with the world *through* that very body in ways fundamentally informed by the body's practical capabilities. This notion of body stresses the duality of human existence as being not merely an object, but also a subject – what I, with Merleau-Ponty (2013) will refer to as a *body subject*. As I will show, Holt's focus on the body as a physical object without reference to the experiencing, acting body subject is what prevents his account from acknowledging the kinds of immediacy between physical and virtual that can characterize esports performance.

To begin with, when examining immediacy in esports performance from the perspective of the subjective body, it no longer suffices to fall back on the assumption that esports is bodily indirect because the body as an object is somehow “stuck” outside of the virtual space. As Osler points out, we conflate the body as an object with the “lived” body subject when we

[…] assume that because we leave behind our physical bodies when we enter online space, we become disembodied. […] More needs to be said to justify the move from saying that the physical, objective body cannot enter online space to saying that the lived body cannot enter online space (Osler, 2021, p. 11).

Likewise, even if an esports practitioner's body is seemingly positioned outside of the virtual space, this does not by itself show that there is not a direct, bodily engagement with said virtual space. Including the body subject as an integral part of our concept of body thus opens a novel route for exploring the place and role of the body in esports, and possibly a way of challenging Holt's argument from immediacy.

### Virtual Spaces of Interactivity

Just as the concept of body subject allows us to think about the body beyond physicality alone, so too it allows us to think about space beyond physicality alone. In this context, whether any kind of technologically mediated interaction really involves a significant spatial difference in terms of domains of execution and application – as Holt (2016, p. 8) insists is the case during, e.g., a telephone conversation – depends on the experienced, bodily interactivity the technology provides or fails to provide for the interlocutors. In this way, the concept of the body subject is bound up with a novel understanding of space beyond physicality alone, namely as a place or field of bodily interactivity.^10^ A space is, on this account, first and foremost a place for a body subject to interact with. This potential for interactivity is not only dependent on the space. As stressed by, e.g., Gibson (2014, pp. 119–135), any perceived environment's potential for interaction, any perceived *affordances* of an environment or space for a subject, will at the same time be intrinsically dependent on the kind of bodily agent engaged with said environment. That is to say, a space and the opportunities for bodily engagement it affords will be contingent on the kind of body subject that engages with it, including the skills and technologies the body subject has learned to capably bring “into his bodily space”, as Merleau-Ponty (2013, p. 146) describes it. For instance, the perceived, bodily significance of a city scape will be profoundly different to a talented parkour runner compared to, for example, a cyclist (Aggerholm and Højbjerre Larsen, 2017). To see what this notion of space amounts to more concretely, consider Merleau-Ponty's description of a football field from the perspective of a body subject:

For the player in action the football field […] is pervaded with lines of force (the 'yard lines'; those which demarcate the penalty area) and articulated in sectors (for example, the 'openings' between the adversaries) which call for a certain mode of action and which initiate and guide the action as if the player were unaware of it. The field itself is not given to him, but present as the immanent term of his practical intentions; the player becomes one with it and feels the direction of the goal, for example just as immediately as the vertical and horizontal planes of his own body (Merleau-Ponty, 2013, p. 146).

Both Holt's account of physical immediacy in sports and Merleau-Ponty's account of a body subject's engagement with their playing field deal with overlaps in the space of the subject and the space of the playing field. However, the difference between what this overlap amounts to for Holt compared to Merleau-Ponty is directly dependent upon their different conceptions of body and space. For Holt, the space of the football field and the space of the footballer is the same, as both the physical body of the footballer and the physical playing field stand in some physically immediate, spatial relation. For Merleau-Ponty, in contrast, the space of the football field and the space of the footballer are the same because of the subjective, bodily engagement of the football player. With a conception of space fundamentally dependent upon the body subject, the question of overlap between domains of execution and application can be reformulated as a question of whether a space succeeds in properly being a space of interactivity for the body subject(s) involved. In terms of esports, the question is if the kind of bodily immediate relationship between practitioner and playing field (as described by Merleau-Ponty above) can be pertinent to esports practice.

Observations of and interviews with high-ranking esports practitioners lend support to the idea that virtual spaces can very much be spaces of interactivity with immediate significance to esports practitioners as body subjects. Esports practitioners have emphasized how not only their physical gaming equipment, but also their mastered in-game virtual abilities become integrated into their sense of body as extensions during performance, shaping their sense of “where” they are when playing; including how they perceive and engage with their virtual space and the other players (Ekdahl, 2021). Moreover, Ekdahl and Ravn (2021), observing and interviewing high-ranking esports practitioners, have highlighted different forms of social, bodily intelligence that talented practitioners develop within the virtual domains themselves as spaces of interactivity. This is something that lets the players read and engage intuitively with each other's virtual body language (e.g., how stressed another practitioner's avatar moves), and even feint emotions and intentions within their virtual domains as an integral part of their performance – just as we might see in purely physical sports. Such descriptions from esports practitioners speak to a rich and complex, bodily engagement with their virtual domains as spaces of interactivity.

The fact that virtuality is an integral part of these practitioners' performance does not have to prevent an immediate overlap between body subject and virtual domain. Rather, as Lindemann and Schünemann (2020) have argued, the relationship between a body subject and a virtual space can be described as a novel form of “mediated immediacy” – not as something fundamentally indirect. In this way, by being attentive to the body subject of esports practitioners, it is possible to challenge the idea that the virtual domains of esports cannot allow for bodily immediacy. One might interject here that by focusing so strongly on the relationship between body subject and virtual space, we seem to have left behind the physical body. Let us consider this in more detail.

### Hybrid Spaces

To push back against the idea that, by focusing on the subjective body, we are leaving behind the physical body, one must keep in mind that the distinction between body as object and subject respectively does not mean that one is somehow separate from the other. Performing esports practitioners are also still physical bodies. While the exact relationship between the body as object and the body subject is an ongoing field of research (Edwards, 1998; Legrand and Ravn, 2009; Taipale, 2014; Liang et al., 2018), in most esports, the virtual engagement of the esports practitioners as body subjects is continuously correlated with the activity of the physical body. This was also clear from our earlier discussion of motor skills, where a constant awareness to and relevance of the physical body were emphasized as key elements of esports performance.

With the concept of the body subject, we need to reconsider how we think about the physical activity of the esports practitioner. On Holt's (2016) account, esports performance is a kind of one-way street transpositionality. Esports practitioners enter physical input to produce a virtual output. As should be clear by now, this account does little justice to the actual experiences and engagement of esports performers, and, indeed, esports practitioners might well be surprised to have their performance described in such a way. No more than we think of a cyclist's pedaling as a separate event from the bike moving forward should we think about the physical input of esports practitioners as a separate event from the virtual activities. This is not to say that physical input and virtual output in esports are metaphysically the same, no more than the cyclist's physical activity is the same as the bike moving forward. The point is that whether it be a cyclist or an esports practitioner, input and output during performance are from the beginning interconnected.

It should also be added that esports often involves complex social settings simultaneously virtual and physical. We see this in the often-favored physically close proximity between players during training and tournaments (Naraine, 2021) so that fist bumping and hugging are a constant opportunity during performance. We also see this in the ways physical body and virtual body of the practitioners can be interconnected socially during performance. Witkowski spells out these forms of hybridity in her fieldwork with actual practitioners of the game Counter-Strike:

In Counter-Strike, the “sporting movement”—needing to see as we move—is achieved by engaging players physically through aspects such as maintaining a controlled body while quickly navigating the environment, by moving the character proficiently with reference to the team (through intracorporeal agility such as “knowing by body” the team tempo), as well as by means of the physicality executed in the muscles and tendons of hands and fingers and in the subtle control of breathing (Witkowski, 2012, p. 359).

None of this is to argue that the physical body literally enters the virtual space, but it is to say that physical body, physical space, and virtual space can be closely interconnected and overlap through the body subject. Alongside the growing intersections between digital and physical space that characterize everyday life for most of us, esports shows us that we may need to start thinking about these intersections as more like “blended” spaces (Krueger and Osler, 2019). Berger (2020) captures this emphatically in his thorough analysis of online games, arguing that, in gaming, embodied subjects synthesize

[…] a single hybrid space, in which the virtual space (constituted under circumstances of virtual presence in the game's finite province of meaning) and the space of the body and its physical surroundings are linked to each other (Berger, 2020, p. 616).

Taken together, this view constitutes a departure from the idea that the physicality and virtuality of esports be fundamentally unconnected separate domains, in favor of an account of esports as jointly physical and virtual, simultaneously interconnected through the subjective body of the practitioner. Both the physicality and the virtuality of esports can, as Hilvoorde and van Pot (2016) emphasize in their analysis of fundamental motor skills in esports, be entwined through the body subject of the esports practitioner.

### Two Objections

Two general objections might be raised against specifically the empirical data emphasized here. First, one might object that, at best, esports practitioners only ever experience the illusion of bodily engagement with their virtual spaces. We find an example of such an objection, with a particular reference to video games, in Fuchs' analysis of empathy and embodiment online (Fuchs, 2014):

The actual melding, however, of body and computer is first introduced via virtual reality in computer games and in cyberspace: no longer a passive spectator, rather transformed into an interactive agent, the user experiences the magical impact of his own activity, and the immersion reaches its maximum level. The illusion of one's own body in motion in the digitally created space favors also the identification with avatars or other surrogates, not to mention the empathetic interaction with virtual persons. One could even speak of an “incorporation” of virtual space (Fuchs, 2014, pp. 66-67).

This incorporation is, however, not a case of real incorporation, according to Fuchs. As Fuchs notes, whereas authentic embodied existence is characterized by elements of resistance and unpredictability, video games are never characterized by these elements as video games always involve a “preset frame” (Fuchs, 2014, p. 170). For this reason, the experiences of embodiment in video games are only illusory. A problem is that, while this notion of a preset frame might fit some types of video games, typically those with a single-player mode (even including the type of esports practice known as speed-running, where a highly predictable game or level has to be finished as quickly as possible through sheer muscle memory), it does not at all capture the movement-, action-, and coordination-complexities of most esports practices. Given that most competitive esports practices are social, and often team-based, elements of resistance and unpredictability are often inevitably at the center of these practices. From very basic movement-related refinements such as particular ways of combining jumping and running to move faster; to new and complex ways of combining and using abilities and weapons in the game-worlds; to, as we have seen, moving and acting deceptively in-game in ways reminiscent of feinting in physical sports (Ekdahl and Ravn, 2021), esports practitioners need to continue to find innovative ways of surprising their opponents, and often even the game designers themselves. It will thus not do to disqualify the authenticity of the embodied experiences of esports practitioners based on a lack of resistance and unpredictability.

Beyond Fuchs' objection, it should likewise be stressed that performing esports practitioners are not somehow confusing their physical body with, e.g., their virtual avatar during performance. A novice gamer might jump back in their seat when shot at in a game, or try to avoid being hit by virtual objects coming at them on a monitor by moving their physical head around, but talented esports practitioners are fully able to perceive the virtual events with the right, hybrid bodily significance (Ekdahl, 2021, p. 360). In a similar vein, it should also be emphasized that esports practitioners are obviously not engaged in some illusion that they are able to physically replicate what happens virtually, which is not to say that what they are doing virtually is not skillful or real (Naraine, 2021).

Second, one could insist that it does not matter what esports practitioners experience nor how they engage with their virtual spaces at all when discussing bodily immediacy. However, while it is certainly possible to deny the relevance of esports practitioners' experiences of bodily engagement when exploring exactly the relationship between esports and bodily engagement, it also seems like an odd thing to insist on. Should only a kind of third-person perspective on esports practice be at all suited to explore the bodily engagement at stake?^11^ After all, in purely physical sports, the experiencing and practical body of the practitioners seems a central element of how and why we appreciate and understand sports. It is not clear why the experiences of esports practitioners specifically should be any less relevant to our understanding of their bodily engagement.

Another, more concise way to respond to this objection is to acknowledge that one might indeed deny the relevance of the practitioners' experiences, and even throw out the entire framework of the subjective body. But, as it stands right now, the discussion of immediacy in esports can either begin (and end) with metaphysical dogmatism about virtuality, or it can begin by looking to the practices themselves prior to such dogmatism. One of these approaches lays the groundwork for a way forward where we might arrive at an at all clearer understanding of the relationship between physicality and virtuality in esports' myriad of practices.

## Limitations, Future Directions, and Practical Implications

A limitation of this contribution concerns the degree to which its critical, analytical approach can stand on its own. For example, in developing a bodily immediate account of esports performance, my critical assessment of Holt has relied on alternative conceptions of, e.g., body and space. While I have tried to justify the relevance of these alternative conceptions, a weakness in relying on them, taken in isolation, is that a one might still reject their validity. However, taken together with the included empirical research with actual esports practitioners, as well as the numerous inconsistencies and the constant danger of circularity highlighted in the indirect account of esports, I have nevertheless provided good reason to be skeptical of the idea that esports entails a necessary gap between domains of execution and application.

In this context, another limitation of this contribution is the relatively narrow amount of empirical research I have been able to draw on. This is, unfortunately, related to the relatively narrow amount of available research altogether that has been conducted on the relationship between virtuality and physicality in the context of esports performance. This speaks to a need for further research that explores the place and role of, e.g., bodily engagement in esports. Future research on esports should, given the account produced here, remain attentive to the first-person, lived engagement of esports practitioners. Moreover, future contributions to esports research should avoid starting out from the assumption that esports be, e.g., indirect image manipulation (Parry, 2019) or in some sense non-physical or unreal. One possible candidate for this is the growing field of research informed by philosophical phenomenology in sports science (Allen-Collinson, 2009; Allen-Collinson and Hockey, 2009; Ravn and Christensen, 2014; Ravn and Høffding, 2017; Heath and Larsen, 2022), with its methodological telos of returning to “the things themselves” (Husserl, 2012, p. 168) prior to our ontological assumptions about them (even if the ‘things’ be virtual).

Lastly, this contribution has implications for practitioners and trainers. In developing an account of esports performance as simultaneously physical and virtual, the study provides a theoretical foundation for the growing research that continues to stress the significance of implementing physical exercise into esports training (Nagorsky and Wiemeyer, 2020; Roncone et al., 2020; Rudolf et al., 2020), and therefore of treating esports as something inherently physical. No less significantly, on the virtual side of things, the account developed here pushes practitioners, trainers, and other esports stakeholders to refrain from treating the virtual spaces of esports in a reductive manner, e.g., as unreal, indirect, or disembodied. Instead, mastering many of the most popular esports games should at the same time be practiced and taught as an embodied process; a process of coming to incorporate a virtual but real space, with its own span of direct, bodily significances.

## Data Availability Statement

The original contributions presented in the study are included in the article/supplementary material, further inquiries can be directed to the corresponding author/s.

## Author Contributions

The author confirms being the sole contributor of this work and has approved it for publication.

## Funding

This research was funded by the University of Southern Denmark (Department of Sports Science and Clinical Biomechanics and Department for the Study of Culture) and the Danish Ministry of Culture.

## Conflict of Interest

The author declares that the research was conducted in the absence of any commercial or financial relationships that could be construed as a potential conflict of interest.

## Publisher's Note

All claims expressed in this article are solely those of the authors and do not necessarily represent those of their affiliated organizations, or those of the publisher, the editors and the reviewers. Any product that may be evaluated in this article, or claim that may be made by its manufacturer, is not guaranteed or endorsed by the publisher.

## Footnotes

^1^I also follow Holt in approaching the general debate around the cybersport thesis in terms of an existential quantifier and not a universal one. As (Holt, 2016, p. 6) notes: “The sensible question is whether videogames *ever* count as sport […]”.

^2^I am aware that shooting as a discipline is a contentious example to use when arguing for something to be a sport. I am not using this example to argue that esports is sports just because shooting might be sports. Rather, I use this example simply to emphasize that we ought to be just as attentive to the whole-body activity of esports practitioners as we might be with non-digital athletes.

^3^Virtual Reality esports.

^4^Augmented Reality esports.

^5^MBVGs, unlike VR, AR and physical esports, have however not received much traction in the broader esports milieu.

^6^Holt's discussion of esports is broader than his focus on physicality alone. However, unless otherwise relevant to the discussion, I here focus on Holt's analysis of physicality and esports.

^7^For the sake of simplicity, I am leaving God out of Descartes' analysis of substances.

^8^One might interject that the camera is translating data into an image only in direct response to the archer's physical movement, but this is not significantly different from what happens in most esports too, where the physical movement of the players' body literally changes their perspective upon the (virtual) domain of application.

^9^The conception of virtual domains in esports as domains of immediate, practical significance for the performing subject is developed further in Section Virtual Spaces of Interactivity.

^10^See Osler and Krueger (2021) for further discussion of such a concept of space in online situations.

^11^For a discussion of the validity of conscious experience as explananda, see Høffding et al. (2022).
